# Ocular effects caused by viral infections and corresponding vaccines: An overview of varicella zoster virus, measles virus, influenza viruses, hepatitis B virus, and SARS-CoV-2

**DOI:** 10.3389/fmed.2022.999251

**Published:** 2022-10-26

**Authors:** Simona Scalabrin, Alice Becco, Alessio Vitale, Raffaele Nuzzi

**Affiliations:** Department of Surgical Sciences, Eye Clinic, University of Turin, Turin, Italy

**Keywords:** virus infection, vaccine, ocular effects, varicella zoster virus, measles virus, influenza viruses, hepatitis B virus, SARS-CoV-2

## Abstract

Many viral infections can affect vision and the visual system. Vaccination to prevent diseases is commonplace today, acting by stimulating an immune response without developing the pathology. It involves the production of persisting antibodies against the pathogen and the activation of T cells. Certain diseases have already been eradicated by rigorous vaccination campaigns, while others are hoped to be eliminated soon. Vaccines currently available on the market are largely safe, even if they can rarely cause some adverse effects, such as ocular complications. Analyzing existing literature, we aimed to compare the pathological effects on the eye due to the most common viral infections [in particular varicella zoster virus (VZV), measles virus, influenza viruses, hepatitis B virus, and SARS-CoV-2] with the possible ocular adverse effects of their relative vaccines, in order to establish a risk-benefit relationship from an ophthalmological point of view.

## Introduction

Vaccine safety represents a very topical theme, especially in the most developed Countries. Some people consider vaccination as unnecessary and potentially harmful, whereas the infectious risk is perceived as irrelevant, often because they ignore the potential harmful effects of diseases. Focusing on the ocular system, we decided to analyze current literature aiming to compare the possible ocular effects caused by different pathogens with those potentially caused by the administration of their related vaccines, to highlight the benefit-risk ratio of vaccination.

Currently, the main pathogens against which effective vaccinations are available are: Varicella Zoster Virus (VZV), Corynebacterium Diphtheriae, Poliovirus, Hepatitis B Virus (HBV), Hepatitis A Virus, Haemophilus Influenzae type B (Hib), Pneumococcus, Meningococcus, Measles Virus (MeV), Rubella Virus, Human Papilloma Virus (HPV), Rotavirus, several Influenza Viruses (IVs), and recently also SARS-CoV-2. In developed Countries, vaccination against these pathogens is largely mandatory or strongly recommended by the competent authorities. In our analysis we will especially focus on pathogens which have been shown to have more frequently serious impact on ocular structures: VZV, MeV, IVs, HBV, and SARS-CoV-2.

## Viral infections and vaccines

### Varicella zoster virus

VZV is a double-stranded DNA virus of the Herpesviridae family (1). Primary VZV infection is responsible for chickenpox, a disease characterized by fever and a typical vesicular rash; it is commonly contracted in childhood, but it can also affect adults, both immunodeficient and healthy subjects (2). The reactivation of the virus, which remains in latent form inside sensory nerve ganglia, causes Herpes Zoster (HZ) (1).

Primary VZV infection in children can sometimes be associated with ocular complications: in 12–25% of varicella cases an acute anterior uveitis, usually mild, can develop, which causes discomfort, lid swelling, irritation, perilimbar injection, photophobia, and decreased visual acuity (3). Generally, this condition is self-limiting and does not cause long-term ocular damage. However, there are rare cases where chickenpox can determine serious forms of ocular involvement. Even in adults, primary varicella infection can become complicated with ocular manifestations, which (3) are usually recurrent granulomatous or non-granulomatous anterior uveitis and keratitis (4). In addition, cases have been reported of primary VZV infection becoming complicated with acute retinal necrosis (ARN) (4).

- Reactivation, causing Herpes Zoster, is a fairly common event (5, 6), but it is more typical in subjects over 50 years and in immunocompromised subjects (7–10) and with immunosenescence due to the aging (11). Herpes Zoster Ophthalmicus (HZO) is the involvement of ophthalmic branch of the fifth cranial nerve, and it represents 10–20% of HZ cases (5).

All ocular structures can be affected by the disease: the virus can affect the eyelids causing hyperemia, edema, skin rash, ptosis, decreased palpebral motility, or even lagophthalmos (the inability to fully close the eyelids) as a result of the paralysis of orbicular muscle (12–14).

At conjunctival level the virus can determine a follicular reaction, with the potential formation of membranes or pseudomembranes; moreover, vesicles can develop on bulbar or eyelid conjunctiva (12–15). It is possible a worsening of the clinical situation with superimposed infections, ulceration, scar development, and eventually symblepharon (a cicatricial fusion between the globe and the inner surface of the eyelid) or mechanical entropion (the inversion or turning inwards of the border of the eyelid against the eyeball, causing the eyelashes to rub against the ocular surface) (12).

The cornea is the ocular structure more frequently involved (about 65% of cases) (12, 15–17): HZO can produce corneal surface epithelial keratitis (12–14), pseudodendrites (10, 13, 14), anterior stromal infiltrates (12, 14), late corneal mucous plaques keratitis (MPK) (18, 19), disciform keratitis (12, 14), endothelitis with potential subsequent loss of endothelial cells (20), neurotrophic keratitis due to involvement of the sensory nerve with corneal hypoesthesia or anesthesia (10, 16, 21), or exposure keratitis, if associated with an eyelid defect (12). In the most severe forms, corneal ulceration and even perforation could appear, generally at perilimbar level (12, 15, 22). Following the acute phase, corneal scars are common and they can manifest as either a stromal haze or opaque areas, eventually associated with corneal thinning (12–15). Finally, in case of extensive corneal involvement, neovascularization can be observed (14).

Other manifestations of HZO are scleritis and episcleritis (13, 15, 23), anterior uveitis (13, 14, 16, 24), cataracts (10) and iris damage with sectoral atrophy (13, 14). As a complication of the uveal involvement there may be iris adhesion to the angle or lens structures, called synechiae (14): in fact it is not uncommon to detect a high intraocular pressure during HZO (10, 13, 15), potentially linked to trabeculitis (14), and secondary glaucoma (10, 13, 25).

Moreover, rare complication of HZO is optic neuritis, which can occur during the acute phase of the infection or as a post-herpetic complication; symptoms include reduced vision and central visual field defects, which are typically associated with edema and hyperemia of the optic papilla in the active phase, and with atrophy of the optic disc thereafter (16, 26–29). Although the actual cause is unknown, it is assumed that optic neuritis can be caused either by direct nerve infection by the virus through the cavernous sinus or by an immune-mediated reaction causing edema of the optic disc and inflammatory demyelination of the nerve (26).

In less frequent cases, HZO can be associated with paralysis of the cranial nerves, especially oculomotor nerve, abducent nerve and trochlear nerve (16, 30–32), and even with retinal detachment (14, 33). As already mentioned, VZV is also the most frequent cause of ARN, a severe occurrence characterized by 1 or more foci of retinal necrosis with defined edges and localized to the retinal periphery, which progress rapidly without antiviral therapy, generally circumferentially (13–16, 33); this pathological condition is a result of occlusive vasculopathy with arteriolar involvement, and is accompanied by reaction in the anterior and vitreous chamber (34, 35). The visual outcome is usually very poor (33), in about half of the cases often less than 20/200 (36), and can be followed by retinal detachment, chronic vitretitis, epiretinal membrane, macular ischemia, macular edema or optic neuropathy (37, 38).

Because of the potential ocular complications, HZO is reported to cause a loss of visual acuity in 6.6–10% of patients (13, 15, 39).

#### Varicella zoster virus vaccines (*Varivax*, *Zostavax*, *Shingrix*)

In many developed countries vaccination against primary VZV infection is recommended in children over 1 year of age, teenagers and adults without a previous history of chickenpox (13, 40); the vaccine name is *Varivax* (or *Varilix*, depending on producer and country), consisting of live-attenuated virus. The incidence of varicella, as well as varicella-related hospitalizations, has decreased significantly since implementation of the varicella vaccination program in 1995. Overall, varicella incidence declined an average of 97% from prevaccine years (from 1993–1995 to 2013–2014) based on data from four states that have been continuously reporting varicella to the National Notifiable Diseases Surveillance System (NNDSS) since before the varicella vaccination program (41).

In very rare cases, anterior uveitis or keratitis have been observed in the days following vaccination. However, in most of these cases, although the temporal correlation, it is impossible to define whether the ocular complications were due to the pre-existing latency of the wild-type virus or to the live attenuated virus injected (40, 42).

There are two vaccinations available for immunization against Herpes Zoster: *Zostavax*, a live attenuated vaccine available since 2006, and *Shingrix*, a recombinant subunit vaccine available since 2017.

Both *Varivax* and *Zostavax* contain the same live attenuated virus, however *Zostavax* contains a higher dose than *Varivax*. Several studies showed that this vaccine significantly reduces (more than 60%) the incidence of herpes zoster and post-herpetic neuralgia (43) and therefore it is assumed that it also reduces HZO rate (14, 43). In the USA it was initially recommended for the population over 60 years old, and later also for the population aged between 50 and 59 (13, 39, 43).

However, some studies revealed that some patients with history of HZO could develop ophthalmic, dermatological or disseminated recurrence following vaccination with *Zostavax* (13, 23, 44).

It was pointed out how *Zostavax*, in order to prevent latent VZV reactivation, increases the activity of cell-mediated immunity, which in some cases can determine a reaction against persistent viral DNA in the eye tissues, triggering keratitis (45–47), sometimes complicated by perforation risk (48), and anterior uveitis (47, 49, 50). In rarer, but more serious cases, ARN following vaccination with *Zostavax* was observed (51–53). Usually, these manifestations were reported to follow vaccine administration for about 2–4 weeks (45, 46, 48, 50, 54). Other studies identify in previous history of HZO a risk factor for a possible recurrence following vaccination (45, 46, 50). On the other hand, reassuringly, a study based on the Health-Claim Database (47) showed that there is not an increased risk of anterior segment complications in patients who received *Zostavax* compared to those who had a first diagnosis of HZ; it was also observed (40, 55–57) that the reactivation of the virus and HZ following vaccination with *Zostavax* is very rare. So, despite these potential risks, the vaccine is considered safe (54), complications are rare (58) and therefore a past history of HZO is not actually a contraindication to vaccination (5, 23, 46, 50, 59, 60). However, particular attention is recommended for these patients, with eye checks in the 4–6 weeks following vaccination (47).

Currently, *Zostavax* has largely been superseded by recombinant zoster vaccine *Shingrix* in many countries. Shingrix is a subunit vaccine that contains a VZV glycoprotein E antigen and the AS01_*B*_ adjuvant system. Shingrix represents a novel, highly effective and well-tolerated vaccine option for reducing incidence of HZ (more than 90% reduction of risk of HZ) and postherpetic neuralgia in adults aged ≥ 50 years. It is not contraindicated in immunocompromised subjects, and it is preferred over a live attenuated HZ vaccine in immunocompetent individuals, according to the US and Canadian guidelines (61).

Post-licensure surveillance of *Shingrix* found a reporting rate for inflammatory eye diseases of 0.6/100,000 with limited reports related to uveitis (62). It is unknown whether any of these patients had pre-existing inflammatory ocular disease, and the possibility that these cases actually occurred from a lack of vaccine efficiency cannot be excluded.

Results from two large randomized placebo-controlled phase 3 trials of the *Shingrix* found potential immune-mediated diseases occurred at a similar rate between those receiving vaccine and controls at all-time points (63). Similarly, subjects with pre-existing possible immune-mediated diseases did not demonstrate an increased risk for a new possible immune mediated process or exacerbation of their prior disease after vaccination compared with controls. Ocular autoimmune diseases were a pre-defined reportable adverse event in both trials; uveitis was only recorded in 1 of 14,645 subjects receiving *Shingrix*.

A single case of ARN and disseminated zoster after receiving the recombinant subunit vaccine has also been described in a 65-year-old woman with past medical history of multiple myeloma (64). Though post-vaccination VZV infection or reactivation appears to be rare, clinicians should be aware of this potential complication to the recombinant subunit vaccine. Nevertheless, as Shingrix does not contain infectious virus, this most likely represented a failure of efficacy in boosting immunity to VZV.

Uveitis recurrence is an infrequent but serious potential ocular side effect of recombinant zoster vaccination. Three cases of uveitis reactivation following Shingrix have been reported: one patient developed a reactivation of a previously controlled multifocal choroiditis within 1 week of receiving vaccine, and two patients with a previously controlled anterior uveitis developed new anterior segment inflammation (65).

The development of HZO following Shingrix is extremely rare, with only two reported cases of HZ keratitis reactivation shortly following the vaccine: an 89-year-old man (66) and a 75-year-old woman (67) with a history of a well-controlled HZO keratitis had developed a recurrent keratitis a few weeks after their first dose of Shingrix. Fortunately, they made a complete recovery following treatment.

The mechanism behind the activation of HZO following the subunit vaccine is unknown. For live-attenuated vaccines activation could possibly come from direct inoculation of the attenuated, but still active, virus strain. For inactivated vaccines such as *Shingrix*, the mechanism is more puzzling; one possible explanation is a general upregulated immune response to the vaccine, causing ocular inflammation. Another hypothesis is that adjuvants contained in the vaccines could create an autoinflammatory response. This phenomena is known as Shoenfeld syndrome (68, 69). *Shingrix* vaccine does not contain aluminum salts (the most common cause of Shoenfelt syndrome), even though it contains a lipid formulation which could represent a possible responsible factor (67).

In conclusion, despite the possibility of ocular effects following Shingrix, they are extremely rare and vaccination is recommended (61, 62). Rather, this report highlights the importance of ensuring primary care providers are aware of a patient’s history of immune-mediated eye disease.

### Measles virus

Measles is an important cause of child morbidity and mortality worldwide; the World Health Organisation (WHO) estimated that approximately 114,900 people, mostly children under 5 years of age, died of measles and resulting sequelae in 2014. The virus causing measles is MeV, an RNA virus belonging to Paramyxoviridae family; it is an extremely contagious virus, which is transmitted by respiratory route (70). Typical prodromal symptoms of measles are fever, generalized malaise, cough, coryza, and conjunctivitis; in this phase the diagnosis can be suggested by the presence of the pathognomonic Koplik spots on buccal mucosa. In following phases the classic maculopapular rash appears, which initially involves the face and then extends to trunk and extremities (71–73). In the most cases, measles is a self-limiting disease and once resolved leaves a permanent immunity. However, exceptionally it can be complicated by severe neurological sequelae such as acute disseminated encephalomyelitis (ADEM), measles inclusion body encephalitis (MIBE), or subacute sclerosing panencephalitis (SSPE) (74).

Moreover, measles can be accompanied by ocular complications: in its prodromal phase, MeV can cause conjunctivitis, characterized by bulbar and tarsal hyperemia with papillary reaction and eventual mucous secretions; corneal involvement, from mild forms such as punctate superficial keratitis to more severe ones such as subconjunctival bleeding, corneal ulceration, corneal perforation, leukoma and, in very rare cases, chorioretinitis and central vein occlusions (75). More rarely, it has been reported optic neuritis, optic atrophy, retinal vasculitis and macular and chorioretinal alterations, such as macular epithelial pigment anomalies, macular edema, macular hemorrhage, internal limiting membrane contracture and serous macular detachment (76–81).

Notably, ocular complications occur in about half of patients affected by SSPE (76, 77) and they may precede, follow or appear simultaneously with other neurological symptoms and signs (78–81). They are often serious and involve the posterior segment, the optic nerve and more generally all CNS structures associated with vision function: symptoms include gradual visual acuity reduction, episodes of transient blindness, homonymous visual field defects, abnormal spatial perception, visual hallucinations, nystagmus, cortical blindness (76–82). The most typical ocular manifestation in the course of SSPE is the appearance of white, focal or multifocal retinal lesions, which rapidly evolve in areas of atrophy of the retinal pigment epithelium and gliotic scars (76–78).

#### Measles vaccine

Measles is best prevented through vaccination: vaccine consists of an attenuated live form of the virus, proved as safe and effective (83). The delivery of the two doses of vaccine needed to achieve a > 90% immunity is accomplished by routine immunization of infants at 9–15 months followed by a second dose delivered before school entry or by periodical mass vaccination campaigns (84). There are only a few case reports in literature about possible appearance of ocular complications related to the administration of this vaccine (85, 86) involving oculomotor palsy and acute bilateral photoreceptor degeneration. Since these are single and sporadic cases, it is not possible to establish a real correlation between the appearance of the ocular manifestation and the administration of the vaccine (86).

### Influenza virus

Influenza type A, B, and C viruses (IAVs, IBVs, and ICVs) are RNA viruses belonging to the Orthomyxoviridae family (87). Among the three, influenza A viruses are clinically the most important, being responsible for severe epidemics in humans and domestic animals. Aerosol droplets transmit the virus, which causes a respiratory disease that can lead to severe pneumonia and even death (88). The main characteristic of IVs is the high variability caused by antigenic shift, which is the result of recombination with other strains. It allows a lack of recognition of the new variant by the immune system. So, IAVs arising from different host species can combine, producing pandemic strains that are antigenically novel but otherwise well adapted to humans (87).

Several case reports shown a correlation between some types of IVs, especially IAVs, and ocular manifestations. Eye complications mainly involve the posterior segment and the uvea. Different kinds of retinitis was correlated with IAVs infection, characterized by submacular hemorrhage without neovascularization (89), macular edema with exudates (90), perifoveal edema with star-like pattern (91) or by alterations visible by fluorangiography, as hyperfluorescent spots associated with multiple dark circular lesions at the posterior pole (92). In some cases (90) angiopathy was observed, sometimes associated with frosted branch angiitis-like fundus (93). In addition, it is occasionally possible to observe generalized uveal involvement, known as uveal effusion syndrome, characterized by conjunctival congestion, tenderness, pain exacerbated by eye movement, choroidal, and subretinal exudation (94). In the majority of cases symptoms resolves completely over time (90, 92–94) even though, in exceptionally severe cases, they can become permanent: an example is reported by Breker et al. (95), describing the case of a 13 years old girl affected by IAV H1N1, who developed encephalitis associated with severe permanent visual impairment, caused by a confluent ischemic retinopathy and infarction of the lateral geniculate body.

#### Influenza virus vaccine

The high mutation rate combined with a high replication speed and possible antigenic shift allow the virus to rapidly change its structural features and so to escape the immune system or become resistant to drugs: this causes annual epidemics (also Influenza type B arising) and demands for compositions of new vaccine (88). The influenza vaccine can be an inactivated virus vaccine or a split-virus vaccine; usually adults receive a trivalent whole virus vaccine, containing three different viral strains anticipated to be prevalent in the upcoming season (96): subjects receiving the influenza vaccine have a reported lower risk of influenza compared with those who do not receive a vaccination over the course of a single influenza season (97).

Some studies have reported cases of optic neuritis following IVs vaccination, with consequent visual field deficits, in some cases recovering after steroid therapy (98–101), but becoming permanent in other ones (96, 102, 103). It has been hypothesized that the involvement of the optic nerve could be triggered by the similarity between some IVs antigens and proteins located in the CNS: the immune response caused by the viral antigen presence would stimulate an inflammatory reaction in the CNS and against the optic nerve, with consequent demyelination (96, 101). Other ocular manifestations following influenza vaccination was observed affecting the retina: these include multiple evanescent withe dot syndrome (MEWDS) (104) and acute posterior multifocal placoid pigment epitheliopathy (APMPPE) (105, 106): also for these manifestations it was assumed that a molecular mimicry between the viral antigens contained in the vaccine and the retinal pigment epithelium (the specialized epithelium lying in the interface between the neural retina and the underlying choroid) would trigger an inflammatory reaction (105). Finally, in rare cases corneal transplant rejection was observed following vaccination, both in case of perforating keratoplasty (107, 108) and in case of deep anterior lamellar keratoplasty (109): however, in these cases the correlation between vaccination and the rejection was vitiated by other factors, which did not make it possible to establish a certain cause-effect relationship.

### Hepatitis B virus

HBV is a DNA virus (110) representing the most common cause of liver cancer in the world (111). HBV mainly infects hepatocytes and during the acute phase causes nausea, vomiting, diarrhea, abdominal discomfort, decreased appetite, fatigue, fever, myalgia, dark urine and jaundice. Interestingly, HBV can also induce ocular manifestations as retinal vasculitis (112), optic neuritis (113–115), paralysis of the third cranial nerve with pupil-sparing (116) and uveitis (117–119). All these manifestations seem to be caused in part by the indirect action of the virus, due to the accumulation of inflammatory debris in different eye structures, and in part by the immune reaction triggered to eradicate the infection (112, 120).

#### Hepatitis B virus vaccine

HBV vaccination significantly reduced the prevalence of the virus worldwide (121). The vaccine is produced through recombinant DNA technique, allowing the administration of the purified HBsAg antigen, usually in three doses generally starting from the third month of life (122).

Reported ocular complications occurring after the administration of the vaccine included uveitis (112–114), which is the most common manifestation and typically appears 3 days or more following the first dose of the vaccine but rarely occurs again after the second and third dose (123, 124). The posterior segment and the optic nerve resulted also potentially involved, with MEWDS (125), APMPPE (126, 127), central retinal vein occlusion (113, 128), papilledema (129) and optic neuritis (113, 130, 131).

### SARS-CoV-2

COronaVIrus Disease 19 (COVID-19), due to SARS-CoV-2, was first reported in Wuhan, China in December 2019 and has rapidly become pandemic all over the world, with an exponential increase in the number of cases. The most common symptoms are fever, cough and fatigue, and sometimes diarrhea. SARS-CoV-2 can be transmitted from person to person mainly through respiratory droplets or close contact (132, 133).

Being the ocular surface exposed to the outside environment, it can be a potential gateway for virus to invade the body. In addition, ACE 2 is a cellular receptor for SARS-CoV2 (134), that can also been detected in human retina, choroid, cornea, and conjunctiva (135–137). For these reasons, the eye is one of the possible targets of the virus, causing a wide variety of ocular diseases. A retrospective study of three hospitals in Wuhan during the very first phase of the pandemic (January 16-February 19, 2020) reported that 1.4% of patients had visual impairment (138). Wu et al. studied the prevalence of ocular manifestations in patients with COVID-19 and reported that chemosis, epiphora, and conjunctival hyperemia were present in one-third of the patients, most commonly in patients with a severe systemic involvement (139).

Moreover, the virus can be present in tear and conjunctival secretions, suggesting that SARS-CoV-2 could potentially be transmitted also through the eyes (140).

Conjunctivitis and keratoconjunctivitis can be the first symptom in infected patients (141). Dinkin et al. also reported two cases of ophthalmoparesis with abducens nerve palsies that developed within a few days of mild respiratory symptoms (142). The abducens nerve controls the lateral rectus muscle, which abducts the eye. Abducens nerve palsy causes an esotropia due to the unopposed action of the antagonistic medial rectus muscle, so the affected eye turns medially and is unable to abduct properly. Chen et al. suggested that ocular manifestations secondary to COVID-19 can also develop in the middle phase of the disease. They reported a young COVID-19-positive patient who developed a bilateral acute follicular conjunctivitis 13 days after illness onset. Viral RNA was detected in conjunctival swabs and RT-PCR was negative at resolution of symptoms (143). Another patient with severe COVID-19 developed a pseudomembranous and hemorrhagic conjunctivitis 19 days after the beginning of symptoms (144).

There is a report about acute corneal endothelial graft rejection with coinciding COVID-19 infection (145). Regarding corneal graft rejection, any systemic immune dysregulation may alterate corneal ocular immune privilege and increase the patient’s susceptibility for rejection (146). Cells of the innate immune system can invade the cornea and result in the up regulation of cytokines and other pro-inflammatory molecules, including tumor necrosis factor–α (TNF–α), and interleukin-6 (IL-6), normally higher during COVID-19 inflammation (147), which can result in rejection of the corneal transplants.

Furthermore, based on murine models of other CoVs, viral-induced retinitis and optic neuritis secondary to autoantibody production against neuroretina should also be possible, so positive patients should be monitored for signs of neuroretinal degeneration in the long term. Hyper-reflective lesions at the level of the inner plexiform and ganglion cell layers have been also described in infected patients (148–150).

#### SARS-CoV-2 vaccine

The European Medicines Agency (EMA) and the US Food and Drug Administration have approved emergency use authorization for several COVID-19 vaccines. BNT162b2 (Pfizer/BioNTech, Mainz, Germany) (151) and mRNA-1273 (Moderna, Cambridge, MA, USA) (152) belong to the category of lipid nanoparticle (LNP)-formulated mRNA COVID-19 vaccines, while ChAdO × 1 (University of Oxford/AstraZeneca, Oxford, UK) (153) and Ad26.COV2.S (Johnson & Johnson/Janssen, New Brunswick, NJ, USA) (154) belong to the category of adenovirus vector COVID-19 vaccines.

Among the adverse events after receiving COVID-19 vaccines, ocular manifestations have been reported, occurring up to 42 days after vaccination and affecting eyelids, cornea and ocular surface, retina, uvea, nerve, and vessels. Vaccine-induced immunologic responses may be responsible (155, 156).

The Pfizer-BioNTech (157) and Moderna (158) vaccine trials suggest an imbalance in the incidence of Facial Nerve palsy following vaccination (1:5,272) compared with the placebo arm of each trial (1:36,938). Nevertheless, based on the odds ratio (OR) from different studies (159), and after adjustment for pre-existing immune- or inflammatory-related disorders, diabetes, and a previous episode of peripheral nerve palsy, it is highly unlikely that Bell’s palsy is associated with COVID-19 vaccination. Reyes-Capo et al. reported that a patient was diagnosed with right abducens nerve palsy (160), while Helmchen et al. reported female with a history of relapsing-remittent multiple sclerosis (MS) who was diagnosed with Optic neuritis with AQP4-antibody negative neuromyelitis optica spectrum disorders-like syndrome 2 weeks after the first dose of the ChAdO × 1 COVID-19 vaccine (161). Maleki et al. reported an old female who had a sudden bilateral loss of vision 2 days after the second dose of BNT162b2 COVID-19 vaccine, and a bilateral Arteritic anterior ischemic optic neuropathy (AAION) was diagnosed (162).

Literature reported six patients with eyelid manifestations after COVID-19 vaccination: eyelid swelling, eyelid purpuric lesions, Herpes Zoster Ophthalmicus (HZO) (163–165). HZO is a result of VZV reactivation, so vaccine may have induced VZV to reactivate.

Six patients developed corneal manifestations after vaccination. The mean age of these patients was 68.5 (range 56–83) years old. The mean duration between COVID-19 vaccination and onset of ocular symptoms was 14.0 (range 7–21) days. The reported ocular manifestations were corneal graft rejection after penetrating keratoplasty (PKP) (166) and corneal graft rejection after Descemet membrane endothelial keratoplasty (DMEK) (167, 168). The activation of the immune system post-vaccination could be a possible involved mechanisms related to vaccine-related corneal graft rejection (146, 147).

Also uvea is reported to be among the ocular structures involved in post-vaccination manifestations, with many new onset uveitis (169–172). Renisi et al. reported an acute anterior uveitis affecting a 23-year-old male after receiving the BNT162b2 vaccine (172). Mudie et al. reported a panuveitis affecting a 43-year-old female who developed decreased vision 3 days after the second dose of the BNT162b2 COVID-19 vaccine, and she was also diagnosed with asymptomatic COVID-19 shortly after the onset of ocular symptoms (171). Goyal et al. reported a 34-year-old man had vision loss 1 week after receiving the second dose of the COVID-19 vaccine, and a bilateral multifocal choroiditis was diagnosed (170). Also one case of acute zonal occult outer retinopathy (AZOOR) (162) and one patient with a reactivation of Vogt-Koyanagi-Harada (VKH) Disease were described: a woman with a pre-existing diagnosis of VKH well controlled for the past 6 years, who manifested a severe reactivation of VKH 6 weeks after receiving the second dose of the BNT162b2 vaccine (173). Furer et al. focused on the immunogenicity of the BNT162b2 vaccine in 686 patients with autoimmune inflammatory rheumatic diseases, reporting one case of uveitis several weeks after the first dose of the vaccine and two cases after the second dose (174).

Similarly retina and its vascularization could be a possible target in post-vaccination ocular manifestations. Four studies (175–178) reported cases of Acute Macular Neuroretinopathy (AMN), a rare retinal disease causing loss of vision, in which a microvascular abnormality in the deep capillary plexus of the retina is hypothesized (179). All patients were female on oral contraceptive pills (OCP), identified as a risk factor for AMN (180), and received the ChAdOx1 nCoV-19 vaccine. Ocular symptoms occurred 2 days after the first dose. The rarity of disease and the temporal association between the vaccination and the onset of manifestations should be taken into consideration, supposing an additional role in AMN pathogenesis of the thrombogenic role of COVID-19 vaccination.

Central Serous Chorioretinopathy has been reported among the possible retinal manifestations following vaccine administration. Fowler et al. reported a 33-year-old male who developed blurred vision and metamorphopsia in his right eye 69 h after receiving the BNT162b2 COVID-19 vaccine (181).

In literature, we found one case of bilateral retinal detachment. Subramony et al. reported a 22-year-old female with myopia who developed vision loss in her right eye 15 days after the second dose of the mRNA-1273 COVID-19 vaccine. Fundoscopy revealed bilateral retinal detachment without any trauma (182).

In addition, 8 cases of Vascular Thrombosis after vaccination are reported, with a mean age of the patients of 42.9 (range 18–60) years old. The mean duration between vaccine administration and onset of ocular manifestations was 8.1 (range 2–13) days. Regarding post-vaccination thrombosis, rare cases of post vaccination immune thrombotic thrombocytopenia and cerebral venous sinus thrombosis (CVST) after administration of the adenovirus vector vaccines ChAdOx1 nCoV-19 and Ad26.COV2 have been well described (183–189). Anatomically, CVST post-COVID-19 vaccination has been reported to occur in virtually all the dural venous sinuses, and a majority of patients are females.

In conclusion, based on the several reported ocular manifestations after COVID-19 vaccination, physicians should be aware of the possible associations between COVID-19 vaccines and ocular symptoms in order to increase early diagnosis and treatment. Considering that COVID-19 vaccines are very recent and that most of the literature includes case reports and series, there may be evolving data on adverse ocular effects of vaccination, and at the moment no certain causality should be established. On the basis that several vaccines (as the ones described in this review) can induce well demonstrated ocular adverse effects, realistically also COVID-19 vaccines can determine ocular complications, both in case of mRNA vaccines and adenoviral vector vaccines. Vaccine-induced immunologic responses could be responsible for the pathogenesis of the ocular symptoms after COVID-19 vaccination (156).

Nevertheless, the overall benefits of the COVID-19 vaccine in preventing COVID-19 are well established, and the incidence rate of ocular symptoms after receiving the vaccine, considering the massive rollout campaign across the world, is considerably lower than the prevalence rate of ocular involvement in infected patients, so people are encouraged to get vaccinated since the benefits outweigh the risks (155). To date, there is no evidence to suggest that individuals should avoid getting vaccinated for ophthalmic-related reasons (156).

## Conclusion and future perspectives

The reported literature shows how ocular involvement may occur as a result of both infection and vaccination (Table 1). Vaccine-associated adverse events such as disease reactivation are uncommon and difficult to prove. In most cases it is not clear if the ocular manifestations (usually described as case reports of few subjects) following vaccination are due to the vaccine itself, and so if they are directly associated with the vaccination, or if they are simply coincidental. It is not possible to exclude that some adverse events would have occurred also in the unvaccinated populations. However, it is clear how the risk of ocular complications following vaccine administration is lower than the potential rate of complications due to wild virus infection.

**TABLE 1:** Reported ocular complications of viral infections and corresponding vaccine.

Varicella zoster virus	
*Primary VZV infection*	Acute anterior uveitis, recurrent granulomatous/non-granulomatous anterior uveitis, keratitis, ARN.
*Reactivation*	HZO: eyelid hyperemia, eyelid edema, skin rash, ptosis, decreased palpebral motility, lagophthalmos, conjunctivitis, superimposed infections, symblepharon, mechanical entropion, corneal surface epithelial keratitis, MPK, disciform keratitis, endothelitis, neurotrophic keratitis, exposure keratitis, corneal ulceration, corneal perforation, corneal scars, corneal neovascularization, scleritis, episcleritis, anterior uveitis, cataracts, iris damage with sectoral atrophy, synechiae, trabeculitis, secondary glaucoma, optic neuritis, paralysis of the cranial nerves, retinal detachment, ARN.

**Varicella zoster vaccines**	

*Varivax^®^*	Anterior uveitis, keratitis.
*Zostavax^®^*	Keratitis, corneal perforation, anterior uveitis, ARN.
*Shingrix^®^*	Inflammatory eye diseases, ARN, uveitis recurrence.

**Measles virus**	
Conjunctivitis with papillary reaction, punctate superficial keratitis, subconjunctival bleeding, corneal ulceration, corneal perforation, leukoma, chorioretinitis, CRVO, optic neuritis, optic atrophy, retinal vasculitis, macular RPE anomalies, macular edema, macular hemorrhage, ILM contracture, serous macular detachment, atrophy of the RPE.	

**Measles vaccine**	
Oculomotor palsy, acute photoreceptor degeneration.	

**Influenza virus**	
Retinitis with submacular hemorrhage, macular edema, perifoveal edema with star-like pattern, angiopathy, uveal effusion syndrome (conjunctival congestion, tenderness, pain exacerbated by eye movement, choroidal and subretinal exudation), ischemic retinopathy.	

**Influenza virus vaccine**	
Optic neuritis, MEWDS, APMPPE, corneal graft rejection.	

**Hepatitis B virus**	
Retinal vasculitis, optic neuritis, paralysis of the third cranial nerve with pupil-sparing, uveitis.	

**HBV vaccine**	
Uveitis, MEWDS, APMPPE, CRVO, papilledema, optic neuritis.	

**SARS-CoV-2**	
Conjunctivitis, keratoconjunctivitis, ophthalmoparesis with abducens nerve palsies, acute corneal endothelial graft rejection, viral-induced retinitis, optic neuritis.	

**SARS-CoV-2 vaccine**	
Facial nerve palsy, abducens nerve palsy, optic neuritis, AAION, eyelid swelling, eyelid purpuric lesions, HZO, corneal graft rejection, acute anterior uveitis, panuveitis, multifocal choroiditis, AZOOR, reactivation of VKH Disease, AMN, CSC, retinal detachment, vascular thrombosis.	

Since sporadic cases are reported, it is not always possible to establish a certain correlation between the ocular manifestation and the administration of the vaccine. AAION, arteritic anterior ischemic optic neuropathy; AMN, acute macular neuroretinopathy; APMPPE, acute posterior multifocal placoid pigment epitheliopathy; ARN, acute retinal necrosis; AZOOR, acute zonal occult outer retinopathy; CRVO, central retinal vein occlusions; CSC, central serous chorioretinopathy; HZO, Herpes Zoster Ophthalmicus; ILM, internal limiting membrane; MEWDS, multiple evanescent with dot syndrome; MPK, mucous plaques keratitis; RPE, retinal pigment epithelium; VKH, Vogt-Koyanagi-Harada.

A future effort should be the development of effective vaccines with less adverse effects, stimulating a protective antibody response and a T cell response without risk of cross-reaction, using as an example purified viral antigens instead of the whole pathogen; however, the main problem in this approach is that the stimulated immune response could not be able to guarantee a lasting immunity comparable to that produced by live-attenuated virus. The key, as already obtained with other pathogens, could be the development of carriers to enhance the immune response triggered by the antigen, without the need to expose the patient to the whole pathogen.

In addition, it would be helpful to develop vaccine to prevent viral diseases with very frequent ocular involvement, such as Herpes Simplex Virus (HSV) infection. HSV is responsible for several distinct medical disorders, such as orolabial herpes, HSV folliculitis, herpes gladiatorum, herpetic whitlow, herpes encephalitis, and eczema herpeticum. Ocular involvement can present as a primary infection or recurrence from latent disease. Almost every ocular structure can be involved, with blepharitis, conjunctivitis, epithelial or stromal keratitis, endotheliitis, iritis, trabeculitis, and retinitis. Ocular HSV infection is usually due to HSV-1, which establishes latency in the trigeminal ganglion, but HSV-2 can also be a cause of HSV keratitis (190). Ocular complications cause significant visual burden, being the most common cause of corneal blindness in developed countries. They also impair quality of life, with need for long-term maintenance medications for recurrent or chronic cases as a basis for effective management (191). Despite this large public health burden, there is still active debate about the optimal management of ocular HSV (192), and the HSV disease remains challenging to prevent, also because the immune-mediated response to HSV plays an important role in physiopathology of herpetic keratitis. Development of an HSV vaccine represents a promising preventing strategy. One exciting area focuses on a corneal dendritic cell based DNA vaccine that has shown encouraging results in murine models (193, 194). In a recent study of mice previously exposed to a live-attenuated HSV candidate vaccine, when challenged with HSV-1, they did not develop any corneal pathology and had complete preservation of visual acuity, with no additional ocular effects (195). If successfully translated into humans, an HSV vaccine would essentially change the management of ocular HSV infection.

In conclusion, the possible, although rare, risk of ocular involvement should not therefore discourage vaccination, which should be promoted and carried out whenever it is deemed useful or necessary. In fact the benefits of vaccines for the patient and for the population far outweigh the risks of the infections, such as possible systemic manifestations and even severe ocular complications.

## Author contributions

All authors gave their substantial contribution to conception and design of the manuscript and to the acquisition and interpretation of data and materials, contribution in drafting the manuscript and in its critical revision for important intellectual content, approved the manuscript in its present form for publication, and agreed to be accountable for all aspects of the work in ensuring that questions related to the accuracy or integrity of any part of the work are appropriately investigated and resolved.
