# Corrigendum: Ocular effects caused by viral infections and corresponding vaccines: An overview of varicella zoster virus, measles virus, influenza viruses, hepatitis B virus, and SARS-CoV-2

**DOI:** 10.3389/fmed.2022.1081686

**Published:** 2022-11-11

**Authors:** Simona Scalabrin, Alice Becco, Alessio Vitale, Raffaele Nuzzi

**Affiliations:** Department of Surgical Sciences, Eye Clinic, University of Turin, Turin, Italy

**Keywords:** virus infection, vaccine, ocular effects, varicella zoster virus, measles virus, influenza viruses, hepatitis B virus, SARS-CoV-2

In the published article, there was an error in the article title. Instead of “Ocular effects caused by viral infections and corresponding vaccines: An overview of varicella zoster virus, measles virus, influenza viruses, hepatitis B viruses, and SARS-CoV-2,” it should be “Ocular effects caused by viral infections and corresponding vaccines: An overview of varicella zoster virus, measles virus, influenza viruses, hepatitis B virus, and SARS-CoV-2.”

In the published article, there was an error. [Keywords: hepatitis B viruses].

A correction has been made to **[Keywords]**. This sentence previously stated:

“virus infection, vaccine, ocular effects, varicella zoster virus, measles virus, influenza viruses, hepatitis B viruses, SARS-CoV-2.”

The corrected sentence appears below:

“virus infection, vaccine, ocular effects, varicella zoster virus, measles virus, influenza viruses, hepatitis B virus, SARS-CoV-2.”

The authors apologize for this error and state that this does not change the scientific conclusions of the article in any way. The original article has been updated.

## Publisher's note

All claims expressed in this article are solely those of the authors and do not necessarily represent those of their affiliated organizations, or those of the publisher, the editors and the reviewers. Any product that may be evaluated in this article, or claim that may be made by its manufacturer, is not guaranteed or endorsed by the publisher.

